# Dividends and Disconnects: Involving Public and Stakeholder Groups in the Development of a Modelling Study Exploring Income Policy and Health

**DOI:** 10.1111/hex.70780

**Published:** 2026-07-30

**Authors:** Olivia K. L. Hamilton, Gillian Fergie, Michal Shimonovich, Fiona McHardy, Daniel Kopasker, Eric Silverman, Peter Craig, Marcia Gibson, Luke Munford, Heather Brown, S. Vittal Katikireddi, Rachel M. Thomson

**Affiliations:** ^1^ Health Evaluation and Health Technology Assessment, School of Health and Wellbeing, Clarice Pears Building University of Glasgow Glasgow Scotland; ^2^ Public Health, School of Health and Wellbeing, Clarice Pears Building University of Glasgow Glasgow Scotland; ^3^ Social Work and Social Policy University of Strathclyde Glasgow Scotland; ^4^ The Poverty Alliance Glasgow Scotland; ^5^ School of Health and Wellbeing, Clarice Pears Building University of Glasgow Glasgow Scotland; ^6^ Division of Population Health, Health Services Research & Primary Care University of Manchester Oxford UK; ^7^ Data Science Institute Lancaster University Lancaster UK

## Abstract

**Background:**

Policy models increasingly guide policy decision‐making. Incorporating public and stakeholder perspectives into modelling processes can produce evidence that is more relevant, appropriate and acceptable to the public. However, public and stakeholder involvement (PSI) in modelling studies is an emerging practice in the field of health inequalities, with little guidance available to support researchers.

**Objective:**

We present a methodological reflection on a PSI process supporting development of a policy model estimating the health impacts of major income supplementation policies. We include a multi‐layered systems map, co‐developed with public and stakeholder contributors, which guided modelling strategy development.

**Methods:**

We engaged 32 people with lived experience of income insecurity and over 60 stakeholders with expertise in socio‐economic determinants of health. Perspectives were gathered through three participatory systems mapping workshops and five policy prioritisation workshops. Workshop contributions were analysed using descriptive thematic analysis. Insights were fed back to the research team, who made changes or additions to the modelling strategy.

**Results:**

We experienced successes and challenges incorporating public and stakeholder perspectives into model development, specifically: engaging people with lived experience of income insecurity; ensuring PSI settings support effective engagement; and appropriately remunerating and safeguarding public contributors. We learned the importance of breaking down methodological silos within research teams to translate rich qualitative insights into actionable modelling outputs.

**Conclusion:**

In data‐driven modelling studies, PSI requires carefully considered design, strong collaboration between team members, and appropriate resourcing to create enjoyable, productive engagement spaces where contributors can safely share experiences and expertise.

**Patient or Public Involvement and Engagement (PPIE) Contribution:**

PSI was central to this work. Our project partners at The Poverty Alliance, a third sector organisation, facilitated involvement of people with lived experience of income insecurity using their internal trauma‐informed approaches throughout all stages.

## 1 Introduction

There is considerable interest among policymakers and public health researchers in major income supplementation policies (MISPs) as a means to reduce health inequalities, as well as to address the increasing cost‐of‐living in many countries. Policymakers have called for actionable evidence on MISPs [1], which we define as policies that bring about a substantial departure from the existing income support system, such as guaranteed basic income schemes, universal basic income policies or conditional cash transfers, but there can be financial, legislative and ethical barriers to conducting trials of new MISPs [2]. One alternative is to estimate the health and economic impacts of new MISPs using policy modelling, which combines available evidence, theory, and data to project health outcomes from policy scenarios. Such models can provide useable evidence [3] and are increasingly used by policy actors to inform decision making [4, 5].

While use of computational models by policymakers is well‐established, acceptability of models to policymakers includes consideration of who participated in model development [6, 7, 8, 9]. A recent scoping review of existing policy modelling research that reported involvement of the public found that very few included studies involved public representatives, and those that did were lacking in reporting detail on who and what they contributed, perhaps reflecting the technical and often opaque nature of the modelling process [10]. Indeed, few health‐focused studies have involved public or stakeholder groups in policy modelling [7, 8, 9], despite a range of potential opportunities and benefits. Public involvement in policy‐modelling may produce evidence that is more relevant, appropriate and acceptable to the public [8] and furthermore, public contributors may derive enjoyment and meaning from their involvement in policy modelling processes [9]. Public and stakeholder involvement (PSI) in modelling studies may have particular value in the field of public health, where stalling progress to reduce health inequalities in the United Kingdom [11, 12] has led to increased calls for further democratisation of the policy‐making process [13]. Policy models that incorporate the experiences and perspectives of public and stakeholder groups into evidence generation improve democratic accountability in social security policy decision‐making—a policy domain that possesses a ‘prevailing democratic deficit’ [14]. Despite these potential advantages of involving publics, alongside professional policy stakeholders, in modelling processes for income‐related policy, several challenges have also been identified, including negotiating power imbalances and maximising inclusivity [10]. Given the dearth of relevant exemplars, contributions to debates on how best to balance these opportunities and pitfalls are warranted.

In this paper, we report our reflections on incorporating public and stakeholder perspectives into a policy model for the economic determinants of health, with a view to identifying broader lessons for researchers conducting modelling studies. The model was originally developed to study employment and income inequalities over time and is being expanded to incorporate a focus on health and health inequalities [15]. This model is now being extended and linked to a new macroeconomic model that considers broader elements of the economy, such as the level of labour demand, in order to appropriately model MISPs. We intend this resource to be used by policy actors to consider policy interventions that are likely to be effective in achieving longstanding health goals.

To achieve this, we involved public and stakeholder groups in several participatory workshops. First, to identify outcome measures of interest and ensure that individual‐level experiences and macroeconomic processes in the model reflect the experiences of people living on low incomes, we co‐created a systems map to illustrate the major pathways through which MISP income could impact health. Second, to inform the selection of policies to be studied using the linked micro‐macro model, we sought public and stakeholder perspectives on the choice of policies and the characteristics of policies (such as who is eligible for the supplement and if there are conditions attached) to be modelled. Alongside providing an account of the development of processes, their delivery and resultant outputs, we reflect on the dividends and disconnects we encountered throughout. By specifying the strengths and limitations of our approach we aim to support future learning towards ‘socially robust policy modelling’ as articulated by Stewart and colleagues [10].

## 2 Methods

First, following the approach of Barbrook‐Johnson and Penn [16], we ran three participatory systems mapping (PSM) workshops (one with public contributors and two with stakeholders), aiming to identify potential causal pathways linking MISP income and health, as well as effect cascades and feedback loops that would be relevant for the modelling of MISPs. The public workshop included contributors with lived and living experience of income insecurity, recruited by a third sector organisation The Poverty Alliance either via their participation bank or their network of public members. The two stakeholder workshops included stakeholders from the Scottish and Welsh governments; an English local authority; third sector organisations focused on health inequalities, families and children, and economic justice; and academics with expertise in income policy.

Second, to inform the choice of policies to be modelled in our future work, we carried out three policy prioritisation workshops with 22 people with lived experience of income insecurity and two further workshops with stakeholders. We sought their perspectives on the MISPs and MISP characteristics that have the potential to impact health. We carried out purposive sampling, including public contributors with a range of employment experiences, living situations, and family structures (further detail available in Appendix S1) and aiming to include stakeholders from each of the UK devolved nations, and with a range of relevant expertise across different sectors. Further methodological detail on the PSM and policy prioritisation workshops is presented in Appendix S1.

**Figure 1:**
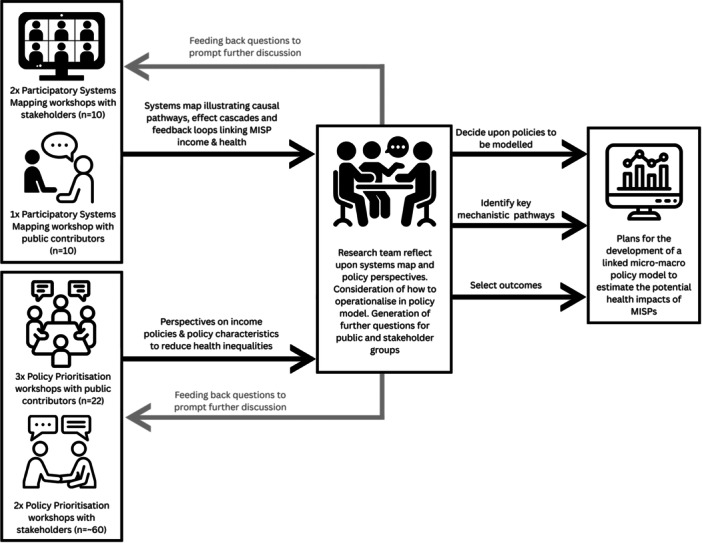
Process for incorporating public and stakeholder perspectives into policy modelling strategy. MISP, Major Income Supplementation Policy.

Public and stakeholder contributions were transcribed by an external professional transcription service. We then carried out a descriptive thematic analysis, noting both common and contrasting themes in the perspectives shared, including which specific policy ideas or characteristics generated most discussion and consensus, and those that were shared by a minority. In doing so, we aimed to draw out unique insights relevant to lived experiences of income insecurity through triangulation between the perspectives of policy actors, academic researchers and people with lived and living experience of income insecurity. Themes that surfaced during our analysis were fed back to the modellers and wider research team via a series of meetings to ensure key pathways for priority MISPs were incorporated into the proposed modelling strategy, to inform the selection of policies to be modelled, and to guide the selection of outcomes (see Figure 1).

Due to the time‐limited and developmental nature of the funding award supporting this work (i.e., resources limited to a 12‐month period), the PSI workshops were one‐off activities, deriving ideas and perspectives to feed into the development of the modelling strategy. Within the Mathematical and Economic Modelling for Vaccination and Immunisation Evaluation (MEMVIE) framework for public involvement in mathematical and economic modelling [8], this would constitute PSI in the earliest stage of the research process (reviewing context and relevance of the model) and should be distinguished from PSI processes that engage public and stakeholder groups throughout the whole research process. In the second phase of our research [17], longer‐term funding is enabling us to involve members of the public throughout the research process including phases of interpreting and reviewing outcomes, decision making and dissemination. In the first phase of the research, described in this paper, we prioritised engagement with members of the pubic with experience of income insecurity, however, longer‐term funding in the second phase is enabling us to engage with a broader range of public groups, including those who are more socioeconomically advantaged, to gain a wider range of perspectives on topics such as revenue raising to support the implementation of new MISPs.

### 2.1 Ethical Approval

As PSI was carried out to inform the development of our modelling strategy, in line with funder guidance, we did not seek ethical approval. This decision was confirmed by the chair of the University of Glasgow Medicine, Veterinary and Life Sciences ethics committee. Third sector organisation, The Poverty Alliance, provided strong collaborative support in line with their own internal approaches to support robust and trauma‐informed approaches to all stages of the engagement.

### 2.2 Findings

In the sections below, we first reflect on the processes of initiating engagement and facilitating the involvement of public contributors in the research process. Next, we present the findings of the PSM and policy prioritisation workshops. Finally, we consider the successes and challenges of establishing a productive dialogue between members of the public, stakeholders and researchers, and of realising the potential of PSI in a single, clearly defined modelling study with limited resources and agreed deliverables.

### 2.3 Initiating Engagement: Involving the Right People in the Right Ways

#### 2.3.1 People

Thirty‐two people with lived and living experience of income insecurity participated in the workshops, supported by our project partners, The Poverty Alliance. Since The Poverty Alliance have established relationships with community members who are typically under‐represented in academic research, we were able to engage with a wide range of people, including those on lower incomes, disabled people, people with caring responsibilities, people from minority ethnic groups, asylum seekers and people who have immigrated or moved to Scotland. Drawing on a wide range of lived and living experiences, reflections from these participants were relevant and valuable to the project, confirming, as others have suggested, the importance of working with trusted community organisations to establish engagement [9]. Furthermore, our third sector partners were also able to comment on experiences of income insecurity that were missing from our dialogues. For instance, those living in rural poverty and the unique challenges that living remotely poses for people who are income insecure.

#### 2.3.2 Setting

While trust and rapport with The Poverty Alliance facilitated public contributors signing up to attend workshops, the workshop settings appeared to impact engagement. Two of the policy prioritisation workshops were held at the University of Glasgow campus (located outside of the city centre in an affluent district dominated by university buildings, museums, parks, Victorian housing and independent retailers) which may have contributed to lower attendance. The start of the on‐campus workshops was characterised by latecomers who had experienced difficulties with public transport and finding the venue. Although several attendees commented that they enjoyed visiting the university, some expressed their unfamiliarity with this part of the city and uncertainty about whether they were permitted to access the campus. In contrast, the final workshop, situated in a community centre in a residential area of Glasgow, was well‐attended from the start, with participants making hot drinks on entry without direction. This familiarity in the community space seems to have had an impact beyond attendance, on participants comfort and ease, likely resulting in better engagement in workshop discussions.

#### 2.3.3 Support and Remuneration

Public members attending and feeling able to contribute at workshops were also contingent upon practical support, such as paying for or providing transport, compensating participants for their time and caring costs, timing workshops appropriately (e.g., to avoid school drop‐off and pick‐up), and selecting accessible venues. The Poverty Alliance's expertise was invaluable across these logistical decisions, for instance, they suggested avoiding scheduling workshops in December when they tend to see an increase in need for support services due to the stresses of the holiday season. However, even with the support of our project partners, remuneration of contributors in receipt of benefits payments poses a particular challenge. The NIHR recommends offering payment in recognition of involvement in the research process (i.e., not for participation as a ‘subject’ in research) and provides a policy laying out rates by activity type [18]. However, although public involvement (or ‘service user involvement’ in terminology used by the Department for Work and Pensions; DWP) in research is not classed as work by the DWP, remuneration, whether in the form of cash or vouchers, is classed as earnings and may affect benefits claims. Large one‐off payments, for example, will lead to deductions from Universal Credit for some claimants if the total exceeds their Work Allowance. Regular involvement in research may also be seen as breaching a recipient's Claimant Commitment and has the potential to affect eligibility for health‐related benefit payments. Additionally, public contributors are often treated as casual employees when being reimbursed, with payments processed through payroll. This has the potential to increase the risk of public members’ contributions being perceived as employment. Despite these risks, clear formal guidance on issues around payment from relevant government departments is lacking [19]. Guided by our project partners, public members were offered a choice of supermarket vouchers of a value in line with NIHR INVOLVE guidelines [18], as an acknowledgement of their contribution. Expenses were also paid for any costs incurred for travel or providing care. The Poverty Alliance were able to provide remuneration directly to contributors, avoiding the complexities of payment via the University system and were able to provide support and advice to avoid any detrimental impacts on income.

#### 2.3.4 Safeguarding

The Poverty Alliance's expertise was also vital in providing adequate time and resource to safeguard members of the public. Given the precarious circumstances of many people living with insecure incomes and the diverse range of lived experiences described in discussions, providing opportunities to debrief in detail with trusted third sector representatives was well‐received by participants. Several made use of time after sessions to talk over issues with The Poverty Alliance staff members, and one contributor was connected with appropriate support services.

### 2.4 PSM Workshops: Public and Stakeholder Perspectives on Pathways Linking MISP Income and Health

**Figure 2:**
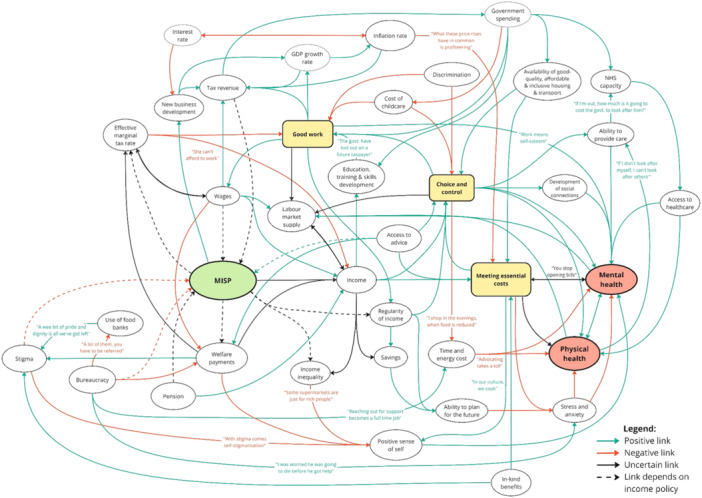
Systems map illustrating hypothesised health impacts of an unspecified Major Income Supplementation Policy (MISP). To read the map, begin with the MISP (green oval) and follow the downstream impacts via the three main pathways (yellow rectangles), to the physical and mental health outcomes (red ovals). Quotations are from public contributors. For further guidance on interpreting the map see Appendix S2. MISP, Major Income Support Policy; NHS, National Health Service.

Over the course of the three PSM workshops, public contributors and stakeholders co‐produced a systems map illustrating the potential causal pathways linking income from an undefined MISP to physical and mental health (see Figure 2). This map illustrates approximately 60 elements and over 150 connections that form a complex network of sources of income, expenditures, individual‐level experiences, and macro‐economic processes.

**Figure 3:**
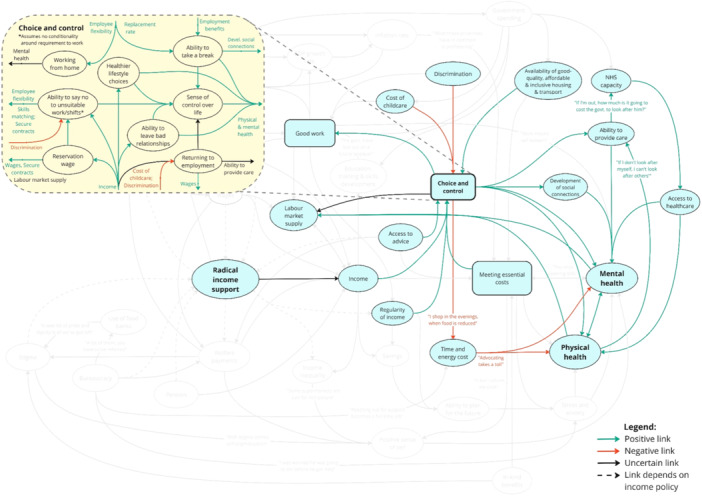
A sub‐set of the full systems map. This sub‐map highlights pathways between income from a MISP and health that are related to choice and control. For further guidance on interpreting the map see Appendix S2. MISP, Major Income Support Policy; NHS, National Health Service.

Public and stakeholder groups identified three pathways as being particularly important in linking MISP income to health: (1) Meeting essential costs; (2) Choice and control; and (3) Good work (presented as yellow rectangles in Figure 2). Although these three pathways are inter‐linked, for ease of presentation they are expanded and presented as separate sub‐maps in Figure 3 and Appendices S3 and S4. Below we discuss one of these pathways as an illustrative example of the insights generated.

Figure 3 illustrates a key theme of both the public and stakeholder workshops that additional income from a MISP would enable recipients to have more choice and control over their lives. Specifically, the types of work they engage with, what they do with their time, and how they spend their money. Stakeholders suggested that the additional income from a MISP might drive up the reservation wage (i.e., the minimum wage rate at which a worker would be willing to accept employment for). They also argued that increasing an individual's reservation wage would enable workers to avoid unsuitable employment and facilitate good work. Additionally, both stakeholders and public contributors suggested that additional income from a MISP might afford recipients more choice over how they provide care for others; the additional income could offset the cost of unpaid caring or contribute to the costs of private care. Public contributors also emphasised how additional income might enable healthier behaviours, such as buying fresh or organic ingredients, or accessing spaces to engage in more physical activity. Contributors noted that these elements would also have a downstream positive impact on mental health by increasing an individual's sense of control over their own life. The emergence of this theme across workshops is broadly aligned with insights from evidence on the psychosocial pathways by which social and material conditions affect health, which highlight control as beneficial to health and lack of control as deleterious [20]. The detailed pathways identified by contributors to our study illuminate these more fully in relation to the specific interventions under investigation (MISPs) and intersections with key social determinants of health.

### 2.5 Policy Prioritisation Workshops: Public and Stakeholder Perspectives on Income Policies and Policy Characteristics to Reduce Health Inequalities

Five policy prioritisation workshops (three with public groups, two with stakeholders) were facilitated (see Appendix S1 for further detail). These involved presenting information on the project and giving examples of income‐related policies (existing and proposed), then facilitating small groups to build ideas and questions before wider whole‐group discussion. For public groups, rather than focus on specific policies, broad questions were asked, for instance ‘What is a policy that would make a difference to you, or those around you?’. In general, these open questions generated a good degree of discussion and attendees expressed interest in modelling a range of policy ideas across three main themes: boosting incomes, reducing costs, and supporting employment. Across all three themes, the ideas that garnered most discussion by both public and professional stakeholders were based on existing policies, rather than innovative or new proposals. As a result, boosting incomes, including discussion of Universal Credit (UC) and Scottish Child Payment (SCP), was the most dominant theme in discussions across all groups.

In relation to boosting incomes, public groups discussed the importance of UC for those on the lowest incomes. Suggested policy refinements varied but extending the eligibility criteria; varying the taper rate for in‐work claimants; and increasing the level were discussed in all public groups. Similarly, among parents who participated, extension of the eligibility criteria for the SCP and increasing the level of SCP were broadly agreed as potentially desirable policy changes for boosting incomes. Boosting incomes via interventions that differ more radically from the current social security system, such as a Universal Basic Income (UBI) were discussed but in less detail by public participants (perhaps reflecting the importance of people's direct experiences in informing discussion). Professional stakeholders expressed more interest in UBI schemes, and related concepts such as Minimum Income Guarantees, as a means of boosting incomes.

Across all groups, policy interventions that reduce costs were also discussed, although these largely received less attention. The exception to this was among public participants who were parents, for whom childcare was a particular priority. In two of the three public focus groups, increased provision and flexibility of free childcare was well supported as a promising policy development. Other policy ideas for reducing costs advocated for by individuals included social tariffs for energy or internet access, or subsidies for everyday essentials, including transport and activities for children. One participant also suggested debt cancellation by local authorities, such as writing off council tax arrears.

Support for good employment was also discussed across all groups as an important aspect of income policy development. A range of suggestions were discussed, with some consensus among public groups around the importance of tailored support into employment, especially through access to training or skills development programmes. However, specific policy ideas or schemes were rarely identified. In relation to wages, there was considerable interest in changes to the National Living Wage, especially increases to the level, recognising the increasing cost of living.

Given the focus in most discussions on refining existing policy designs, more in‐depth PSI (two or three stages) might have asked exploratory questions about *types* of policies followed up with a rapid evidence review of international policy options to provide a wider range of opportunities for comment. Alternatively, some have suggested the potential of health researchers adopting a more structured process like Levitas’ Utopia as Method to free up thinking around policy options [21].

There was a general consensus within and between public and stakeholder groups that certain characteristics of income policies were particularly impactful for health. A common suggestion was that these characteristics could be varied in the modelling to create a range of comparable policy scenarios ranging from minimal, to more radical systemic change. These aspects included, for example, variation in the monetary value of the income supplement, which could be increased in line with inflation or with an estimate of the cost of living, or seasonally, to cover increased fuel costs; variation in the value of the supplement depending on recipient needs and circumstances, and the source of financing, which could include increasing taxation on wealthier individuals, or corporations, or sequestering funds that are retained by individuals via tax loopholes.

However, there were also some areas of disagreement. While some attendees were in favour of a universal income support scheme, under which everybody would receive the same amount of income, others objected to this, suggesting that universalism does not work for people with additional costs related to their needs and circumstances, such as disability, or living with a large family. While most contributors were not in favour of conditions being applied to income support, some members of the public suggested that conditions to incentivise MISP recipients to come off income support and into good work might be beneficial. Additionally, among public contributors there was some (often tense) disagreement around the scope of eligibility for income support, with a small number suggesting that individuals who engage in negative health behaviours should not benefit. As discussions progressed, this perspective seemed to shift as contributors considered more macro‐level determinants of health and focused less on individual behaviours.

Workshop attendees also discussed the kinds of outcomes that future modelling work should examine, which included health‐related outcomes (such as life expectancy, healthy life years, and in particular, mental health), educational outcomes, and employment outcomes (including take‐up of training opportunities, entry into good work, and employment duration). Attendees also expressed an interest in modelling the differential health impacts of MISPs on priority family groups as defined by the Scottish Government [22], people with disabilities, children with additional needs and their parents, people who are out of work or in unsatisfactory work, low‐income households, and people seeking asylum.

### 2.6 Surfacing Public and Stakeholder Perspectives and Incorporating Them Into the Policy Modelling Strategy

As noted, the primary aim of PSI was to inform the development of an open‐source policy model. Discussing this aim and policy modelling more broadly with groups of public non‐specialists generated several challenges. First, although public contributors were motivated to take part in discussions by a broad interest in income policy and health, researchers were conscious of establishing clear expectations that, at this stage of the research, contributors’ influence was limited to informing a model for generating evidence on policy, rather than directly influencing policy itself. This transparency was particularly important for some public contributors who were used to exerting influence on policy actors directly through their anti‐poverty advocacy activities. A further challenge for researchers was communicating sufficient methodological information about policy modelling processes to ensure contributors were informed about what they are contributing to, while maintaining accessibility and not overwhelming them with technical details. In time‐limited engagements with public and professional stakeholders the researchers did not wish to spend the majority of time in sessions explaining concepts and consequently establishing themselves as ‘experts’ when the purpose of the encounter is to listen and learn from those with lived experience or contextual policy expertise. In these challenges, The Poverty Alliance guidance was invaluable and although the balance was difficult for researchers to gauge, few participants fed back that they were overwhelmed.

Public and stakeholder perspectives were fed back to the research team at a series of meetings in which modellers considered how they could be incorporated into the modelling strategy. Some of these considerations were relatively straight forward. For example, the systems map shared several features with the team's existing microsimulation framework [23] (e.g., the relationship between the reservation wage and labour market supply), confirming that these elements were appropriately represented in the policy model. Incorporating other elements of the map into the modelling strategy will require relatively minor methodological developments. For example, the research team is considering adding processes that better reflect elements of the *meeting essential costs* pathway (see Appendix S3), using data from Understanding Society [24] on household finances. To incorporate elements of the *choice and control* pathway, the team is also considering ways of incorporating data from the Recovering Quality of Life questionnaire, which includes relevant questions [25, 26].

Perhaps inevitably, the incorporation of some elements from the systems map requires substantial methodological development. For example, stigma associated with being a benefit recipient was described by public contributors as having a profound impact on mental health. This prompted a review of data sources on experiences of stigma by the research team. There were also elements of the map that could not be modelled directly within the scope of our current project (e.g., experiences of bureaucracy in the benefits system), owing to the complexity of their relationships with other elements and/or lack of availability of appropriate data. Similarly, elements such as discrimination are experienced differently by certain groups of individuals and would rely on data relating to some of the most marginalised populations, which are not well covered in even the highest quality datasets relied on for the modelling (e.g., asylum seekers; people experiencing homelessness). It is important to acknowledge, however, that it is not desirable to model all pathways identified in the systems map. It is a well‐established principle that models should be as simple as possible while still being able to answer the questions of interest. Modelling studies, just like much other research, require achieving a balance between granularity and actionable insights, with the choice of pathways to be modelled dependent on the policy focus of the model. Considering how contributor experiences and perspectives are incorporated into models helps to stress test the model's underpinnings, however, for the research team and others considering this methodology, it also raises questions about how best to take forward the rich insights that we have not been able to incorporate into the modelling strategy. On some occasions, this may involve incorporating some elements into the model indirectly (e.g., allowing for adverse impacts of being a benefit recipient beyond a lack of income) while on others it may highlight model limitations that should be transparently communicated (e.g., the stigmatising nature of interactions with the welfare system).

Certain policy ideas that arose from the policy prioritisation workshops were considered more challenging to model within the context of existing and expected funding calls. Although of interest and potentially modellable, the health impacts of policy ideas involving indirect income support, such as universal free childcare, for example, would require substantial methodological development beyond the scope of the current project. This idea and others, including more tailored employability services and social tariffs for utility and internet services, were therefore considered but not immediately developed by the research team, despite being supported at the public workshops. To support public contributors in making more immediately operable policy suggestions, the research team have identified a need for closer integration between researchers carrying out the PSI work and the modelling development work, and a more in‐depth understanding of one another's methodologies. For instance, PSI researchers would have benefitted from a clearer understanding of the capabilities and limitations of the modelling process in order to set realistic boundaries around the more ‘radical’ policy ideas that were discussed at the workshops and produce more actionable outputs for the modelling team. Similarly, the modelling team would have benefitted from greater input into to the planning, preparation, and where possible, conduct of the PSI activities.

## 3 Discussion

In this paper, we have reflected on the successes and challenges of incorporating public and stakeholder perspectives into a strategy to develop a simulation model to estimate the potential health impacts of MISPs. A vast literature reports experiences of PSI in various domains of health research, including health inequalities, health economics and health policy [9, 27, 28, 29] and tools exist that describe best practices for the involvement of public contributors [27, 30]. Within this literature, factors such as communication, inclusivity and representation, transparency, support for participation and practical arrangements are commonly highlighted as areas of opportunity and of challenge. This was confirmed by our experience; however, we found that the somewhat abstract, data‐driven nature of our policy modelling project demanded even greater consideration of these elements. A summary of lessons learned from our PSI process is presented in Table 1, forming a preliminary set of key considerations and working recommendations for the next phase of our study. We also welcome use (alongside established standards for public involvement in research [32]) and further development of these recommendations by other researchers wishing to embed PSI in modelling research.

**Table 1:** Key considerations and working recommendations for future PSI in modelling studies.

Key consideration	Working recommendation for future PSI
How to engage safely and respectfully with communities of interest?	Consider partnering with an organisation that has trusted working relationships with communities you would like to include. They may be able to support with reaching community members and advise on methods of engagement, logistical decisions, and safeguarding processes. Partner organisations should be involved from project outset and be included in grant proposals, where appropriate. Partner organisations should also be appropriately remunerated, including administrative costs (e.g., time to attend study meetings).
How to establish a welcoming and inclusive ‘deliberative space’?	Contributors should be remunerated for costs incurred, including travel costs and the cost of professional care cover for children or adult dependents (see consideration below). Wherever possible, schedule engagement around the needs of contributors (e.g., for parents, avoid meeting outside of school hours or in school holidays). Hold in‐person events at ideally familiar, accessible venues and consider any potential power dynamics of hosting contributors on the university campus.
How to remunerate contributors appropriately?	Contributors should be remunerated appropriately for their involvement in the research process. However, be aware of potential adverse consequences when compensating benefit recipients, who may experience deductions to benefit payments. Be prepared to support potential contributors with this prior to their taking part. See NIHR's Public Contributor Reward and Recognition Policy [18] and NIHR's Payment Guidance for Members of the Public Considering Involvement in Research, for information [31]. Plan practically for how to reimburse contributors, as University payment systems can be challenging to navigate and can introduce delays.
How to support members of the public to engage meaningfully?	Manage expectations regarding what the PSI process aims to achieve, especially around impact on policy or practice. Consider the balance of contributions from facilitators and public and stakeholder contributors, avoiding establishing facilitators as ‘experts’ and allowing ample time for contributors to voice and discuss their perspectives. Seek advice from partner organisations or appropriate public panels on accessibility of workshop materials.
How best to incorporate PSI perspectives into modelling strategy?	Ensure PSI facilitators understand the windows of opportunity for informing modelling strategy (i.e., which aspects of the model can and cannot be altered or amended). Involving members of the modelling team in the development of the PSI activities might help to ensure that activities surface actionable insights, as opposed to those that require developments beyond the scope of current model capabilities.

A key reflection on our PSI process concerned the value and importance of our partnership with The Poverty Alliance. Having a project partner's time (both research and administrative) fully costed into a research grant is instrumental to enabling them to support safe and productive PSI. The Poverty Alliance have well‐established and trusted relationships with community organisations and individuals in the local area and were able to facilitate reaching individuals with lived experience of income insecurity. Their guidance on practical elements of workshop organisation, such as workshop location, communications and remuneration was also invaluable. Such practicalities are fundamental to establishing a welcoming and inclusive ‘deliberative space’ [8] that supports participatory engagement, especially in technical, data‐intensive research and as such, should be considered intellectual choices [9, 30]. Reflecting on their engagement with members of the public in the SIPHER Consortium policy modelling programme [33], Stewart et al. [9] note that lack of attention to these choices can have negative repercussions not only for engagement with the project, but more importantly, for how public contributors feel about themselves and their own lives.

Our experiences highlight that researchers engaging with people on low incomes need to understand the potential repercussions of remunerating benefit recipients and to be able to support public contributors to find out how receipt of payment might affect their own personal circumstances. While some funders provide guidance on the interaction of public involvement in research and social security regulations, the onus is on members of the public to ‘seek expert, personalised advice before accepting payment for involvement’ [31]. The fact that these issues remain unresolved despite increasing expectations from research funders to embed public engagement and involvement (PPI) within research, suggests that the exclusion of the most financially vulnerable individuals in our society is ‘baked‐in’ to many PPI processes. One (somewhat unsatisfactory) solution is to consult with public contributors on alternative forms of reward (e.g., offering desired educational or training courses) for commitment of their time, experience and expertise to the research process, in cases where they are unable to accept financial compensation. Training for DWP staff on public involvement and engagement in research may additionally ensure that NIHR's definition of ‘service‐user involvement’ in research is appropriately distinguished from employment, to avoid negative financial repercussions for benefit recipients.

Involving groups of public non‐specialists in a policy modelling study with relatively abstract, research‐focused aims generated challenges of establishing boundaries and managing expectations, of succinct and careful communication, and of establishing genuine two‐way dialogue between stakeholders and research teams. Recognising that members of the public contributing to research are often motivated to achieve ‘real world impact’ [9], we managed expectations regarding what our PSI process was designed to achieve to avoid disconnects between perceived and actual outcomes of the research [34]. Ultimately, stakeholders and members of the public were motivated to contribute and engaged well with what we asked of them, resulting in a wide range of perspectives that were informative for our modelling strategy. In particular, the co‐produced systems map was effective in ensuring that key causal pathways between MISP income and health are represented in the modelling framework. PSM workshop discussions also identified relevant outcome measures for the modelling and pushed researchers to consider alternative datasets and methodological developments required for more complex policy scenarios.

Public and stakeholder input into model development has the potential to ‘ground models in the reality of lived experience and behaviour’, enhancing their ecological validity [8]. The systems mapping successes described above go some way towards achieving that. Whereas it is not desirable to explicitly model all relevant experiences described by contributors, the PSM and the policy prioritisation workshops surfaced a significant number of perspectives and suggestions that could not currently be incorporated into the modelling strategy either due to a lack of available data or due to the methodological complexity of the modelling required. While this is unavoidable in a process that seeks to distil the richness of lived experience and expertise into a policy model designed to generate quantitative outputs, some adaptations to our PSI process may have helped. First, public contributors may have benefitted from more information about the social and economic determinants of health inequalities as a framework for the policy prioritisation discussions. This may have aligned idiosyncratic and often shifting perspectives on the root causes (and thus potential policy solutions) of health inequalities, which at times focused on individual behaviours rather than on upstream determinants of health [34, 35]. Second, it may have been possible to guide public and stakeholder contributors to identify policy ideas that could more easily be operationalised in the policy model by breaking down siloed ways of working between the PSI and modelling teams. On the part of PSI researchers, a greater understanding of policy modelling processes (e.g., how experiences and perspectives are operationalised, the requirements of datasets needed do this, methodological constraints of modelling complex relationships) could help guide contributors in the identification of potential policy scenarios that could feasibly be modelled. Contribution of expertise from the modelling team during the design of the PSI activities could also support achievement of that fine balance between supporting contributors to safely and meaningfully express their experiences and to share perspectives that can more readily feed into the modelling process. Within SIPHER [33], this disconnect was addressed through training for the PSI and modelling teams in one another's respective methodologies.

While the full breadth of public and stakeholder perspectives was not incorporated into the modelling strategy, an array of pathways, perspectives and concepts have informed it and have demonstrated additional relevance beyond the current study. The systems map generated has already been shared and widely used by a range of academic and policy audiences, confirming the potential of well‐crafted systems maps for conveying complex information in relatively digestible forms as outputs in themselves. This highlights the potential value of presenting some of the richness of qualitative perspectives, that cannot be modelled, in complementary outputs that can be presented alongside modelling results. Recognising the context in which these outputs emerged and with appropriate ethical permissions, such outputs might also be useful for researchers interested in developing similar projects, reducing the burden on disadvantaged communities to contribute to multiple similar research studies [36].

## 4 Conclusions

In the current study, public and stakeholder contributions pushed researchers to carefully consider model underpinnings, have resulted in the inclusion of alternative or additional pathways, and have prompted ideas for future methodological development. Designing PSI processes, especially in the context of data‐driven modelling studies, is labour intensive, intellectual work and must be appropriately resourced to create an enjoyable and productive deliberative space in which contributors can safely share their experiences and expertise. Crucially, close collaboration and mutual methodological understanding between team members conducting the PSI and the modelling work is needed to establish a dialogic process capable of translating rich experiential insight and expertise into meaningful model developments.

## Author Contributions

**O.K.L. Hamilton:** conceptualisation, methodology, investigation, writing – original draft, writing – review and editing, data curation, formal analysisb. **G. Fergie:** investigation, methodology, writing – original draft, writing – review and editing, data curation, formal analysis, conceptualisation. **M. Shimonovich:** writing – review and editing, investigation. **F. McHardy:** conceptualisation, methodology, writing – review and editing, investigation. **D. Kopasker:** writing – review and editing, investigation. **E. Silverman:** writing – review and editing, investigation. **P. Craig:** investigation, writing – review and editing. **M. Gibson:** investigation, writing – review and editing. **L. Munford:** investigation, writing – review and editing. **H. Brown:** investigation, writing – review and editing. **S. V. Katikireddi:** conceptualisation, investigation, writing – review and editing. **R. M. Thomson:** conceptualisation, investigation, writing – review and editing.

## Conflicts of Interest

The authors declare no conflicts of interest.

## Supporting information


Supporting File

## Data Availability

Data sharing is not applicable to this article as no datasets were generated or analysed during the current study.
