# Supplementary material for: Dividends and Disconnects: Involving Public and Stakeholder Groups in the Development of a Modelling Study Exploring Income Policy and Health
Source: Health Expect. 2026 Jul 30;29(4):e70780. doi: 10.1111/hex.70780 (PMC13421790; doi:10.1111/hex.70780)
Supplement: Supplementary file 1 — Supporting File [file HEX-29-e70780-s001.docx]

**Appendices**

[Appendix 1: Further information on the Participatory Systems Mapping and Policy Prioritisation Workshops 2](#_Toc216627005)

[Appendix 2: Guidance on interpreting the systems map 5](#_Toc216627006)

[Appendix 3: Meeting Essential Costs sub-map 6](#_Toc216627007)

[Appendix 4: Good Work sub-map 7](#_Toc216627008)

### Appendix 1: Further information on the Participatory Systems Mapping and Policy Prioritisation Workshops

**Participatory Systems Mapping Workshops**

Following the approach of Barbrook-Johnson and Penn^[[1]](#footnote-1)^, we ran three participatory systems mapping (PSM) workshops (two with stakeholders and one with public contributors), aiming to identify causal pathways linking MISP income and health, as well as effect cascades and feedback loops that would be relevant for the modelling of MISPs. The first workshop was held in-person with ten people with lived experience of income insecurity. Attendees had a range of employment experiences (including being in-work, out of work, and retired), living situations (including living alone, living with others), and family structures (such as having children, not having children, and being informal carers for family members or friends). Many of the attendees had experience of claiming benefits in the UK and several acted as advocates for others facing financial hardship. Several attendees were either seeking asylum or had been granted refugee status in the UK.

At the workshop, facilitators first presented a primer on MISPs and an overview of the PSM process. In small groups, attendees then discussed and recorded responses to the following questions on sticky notes, which were then used to create the systems map with guidance from facilitators: *1) In what ways would a scheme to increase your income make a difference to your life? 2) In what ways might that impact your physical and mental health?*

We ran two subsequent workshops online with ten stakeholders, including four stakeholders from the Scottish and Welsh governments, one from an English local authority, three from third sector organisations focused on health inequalities, families and children, and economic justice, and two academics from universities in Scotland and England. The first stakeholder PSM workshop followed a similar format; in break-out groups, attendees discussed the following question: *In what ways might a radical income policy impact physical and mental health?* Attendees recorded their responses on Padlet ([www.padlet.com](http://www.padlet.com)) before feeding-back to the overall group and guiding facilitators to build the map using MIRO ([www.miro.com](http://www.miro.com)). Draft systems maps from the lived experience and stakeholder PSM workshops were then integrated by University of Glasgow researchers, before being presented to the same group of stakeholders at a further workshop. At this final workshop, attendees carried out validation of the map, discussing the major pathways, and elements to be adapted, added, or removed.

**Policy prioritisation workshops**

To inform the choice of policies to be modelled in our future work, we carried out a series of policy prioritisation workshops with public and stakeholder groups, seeking their perspectives on the MISPs and MISP characteristics that have the potential to impact health. We conducted three workshops with twenty-two people with lived experience of income insecurity, who were recruited by The Poverty Alliance. The second workshop specifically invited contributions from priority family groups as defined in the Scottish Government’s Child poverty strategy, which includes minority ethnic families, lone parent families​, families with a disabled person​, families with three or more children, families with a child under one year​, or families with mothers aged twenty-five or younger. The third workshop specifically invited contributions from people who were in unsatisfactory work (low paid, insecure, low quality), or who were unemployed. Across the three lived experience workshops, most attendees were in receipt of social security payments, including Universal Credit (UC), the Scottish Child Payment (SCP), Child Disability Payment, or Carer’s Allowance, with several attendees in receipt of multiple payments. Around half of the attendees were either currently seeking UK asylum status or had been granted asylum status in the past.

Additionally, we conducted two further policy prioritisation workshops: the first was online with the same 10 stakeholders who attended the PSM workshops, and the second was with approximately 50 stakeholders at the Poverty Alliance annual conference 2023, including representatives from local authorities, national and local third sector organisations, philanthropic organisations and healthcare professionals.

At these workshops, facilitators presented a primer on policy modelling before posing one of the following questions: 1) What is a policy that would make a difference to you, or those around you? (for people with lived experience), or 2) What is a policy that would make a difference to people with low income (for stakeholders)? Attendees were given fifteen minutes to discuss this in small groups, before presenting responses back to the wider group. Facilitators then guided attendees through an activity to design an income-boosting policy by expressing their preferences around different policy characteristics. We developed a set of policy characteristic question prompts (see below table) and attendees were invited to record their responses on sticky notes/Padlet, before discussing them as a group.

All workshops took place between June and November 2023 and were co-facilitated by researchers from the University of Glasgow (OKLH, MSh, GF) and The Poverty Alliance (FMcH).

Discussion prompts relating to MISP characteristics, presented to attendees at the policy prioritisation workshops

| **Aspect of policy design** | **Discussion prompt** |
| --- | --- |
| Eligibility | Who would be eligible? Would it be for everybody, or just for certain people? If it’s just for certain people, how would they be chosen? For example, by the number of people in their household, how much they earn, etc? |
| Conditionality | Would there be any conditions? For example, you only continue to get it if you do certain activities like looking for work or driving your car less. |
| Level | How much would people receive? How much money would be needed to make a positive difference to people's lives? Would everybody get the same? If not, how would we decide who gets what? |
| Form | What form would people receive it in? A bank transfer? Vouchers? |
| Variability | Would the amount always be the same? Or would it change over time? |
| Frequency of payments | How often would people receive it? Every week? Every two weeks? Monthly? Yearly? A one-off? |
| Withdrawal mechanism (i.e. taper rate) | If people came off the scheme, how would that work? Would payments stop suddenly, or reduce little by little? |
| Financing | How would it be paid for? For example, by raising income tax, taxes on large companies? |
| Duration | How long would it last for? Is it protected by law, so would last indefinitely? Or would it last only for a set amount of time? |

### Appendix 2: Guidance on interpreting the systems map

| **Feature of Systems Map** | **Interpretation** |
| --- | --- |
| Ovals | Ovals represent different elements of the system. These can be thought of as variables that can increase and decrease. |
| Arrows | Arrows represent causal relationships between elements. The colour of the arrow indicates the nature of the relationship. Red arrows indicate a negative relationship; green, a positive relationship; and black arrows indicate a relationship that could be either positive or negative, or one that is unknown. The terms positive and negative are used to indicate a hypothesised statistical relationship (i.e., a 'positive' relationship indicates that as one element increases, so too does the other) as opposed to indicating a value judgement on whether one element might have a ‘good’ or a ‘bad’ impact on another. |
| Dotted arrows | Dotted arrows indicate relationships that depend upon the nature of the characteristics of the radical income policy. For example, if the income policy were to be universal, there would likely be no stigma around receipt of the income support payment, therefore the arrow linking the stigma and radical income support elements is dotted. |
| Quotations | The map includes quotations from people with living experience of income insecurity who attended workshop 1. These quotations are presented to illustrate the thinking behind some of the links and to incorporate their lived experiences into the map more explicitly. |
|  | |

### Appendix 3: Meeting Essential Costs sub-map


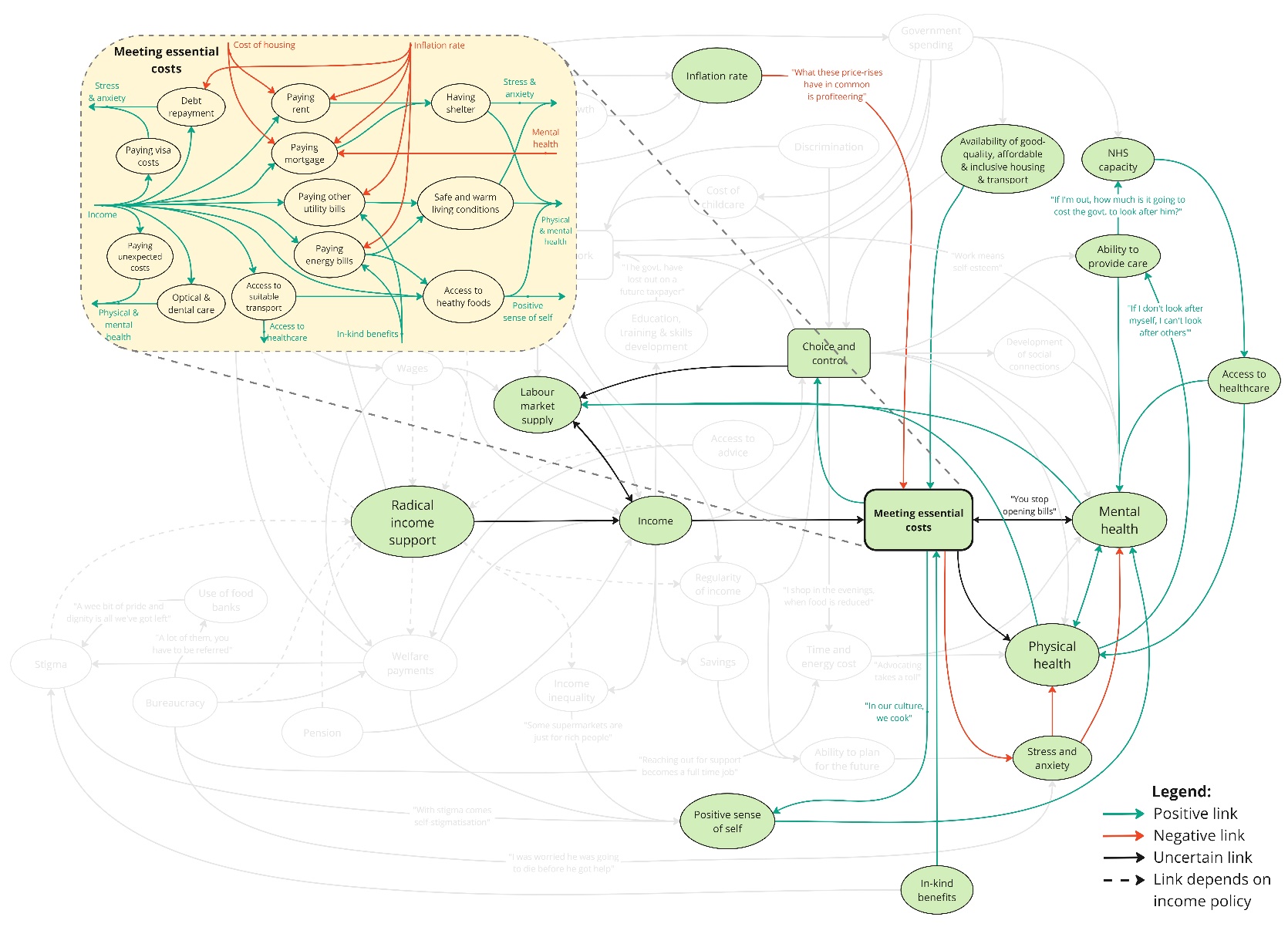
A sub-set of the full systems map presented in Figure 2 in the main text. This sub-map highlights pathways between income from a MISP and health that are related to meeting essential costs. MISP = Major Income Support Policy.

### Appendix 4: Good Work sub-map


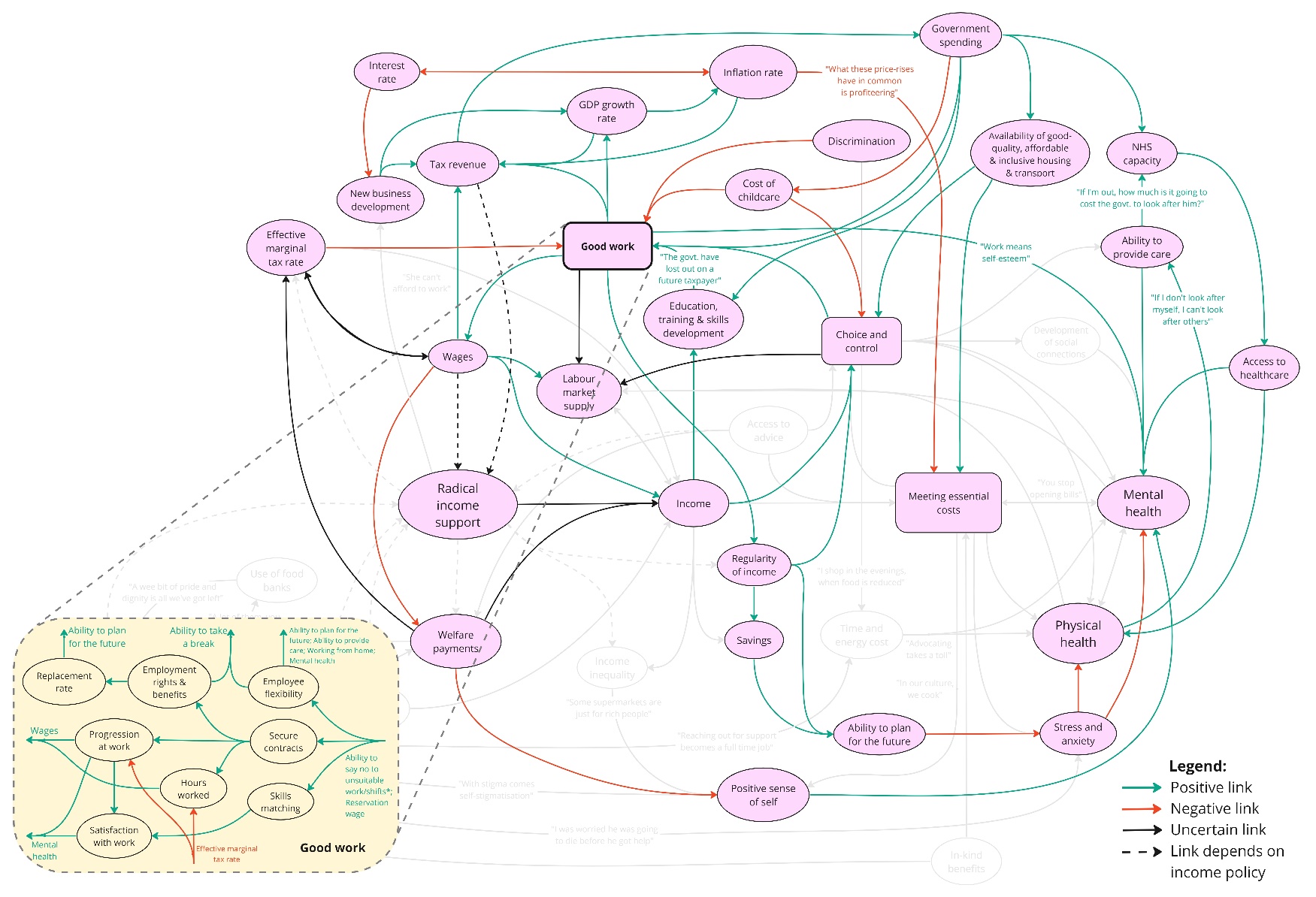
A sub-set of the full systems map presented in Figure 2 in the main text, highlighting pathways between income from a MISP and health that are related to good work. MISP = Major Income Support Policy.

1. Barbrook-Johnson P, Penn A. Systems Mapping: How to build and use causal models of systems: Palgrave Macmillan; 2022. [↑](#footnote-ref-1)
